# Activity-related pain and sensitization predict within- and between-person pain experience in people with knee osteoarthritis: An ecological momentary assessment study

**DOI:** 10.1016/j.ocarto.2024.100439

**Published:** 2024-02-10

**Authors:** Mark Overton, Nicola Swain, Carrie Falling, David Gwynne-Jones, Roger Fillingim, Ramakrishnan Mani

**Affiliations:** a Centre for Health, Activity and Rehabilitation Research, School of Physiotherapy, University of Otago, New Zealand; b Department of Surgical Sciences, Otago School of Medicine, University of Otago, New Zealand; c Pain Research and Intervention Center of Excellence (PRICE), Department of Community Dentistry and Behavioural Science, University of Florida, USA

**Keywords:** Knee osteoarthritis, Pain, Sensitization, Movement evoked pain, Ecological momentary assessment

## Abstract

**Background and objectives:**

Knee Osteoarthritis (OA) is a prevalent musculoskeletal condition that often results in pain and disability. Determining factors predicting variability in pain experience is critical to improving clinical outcomes. Underlying pain sensitization and its clinical manifestations, such as activity-related pain, may better predict the knee OA pain experience. This study aimed to determine whether Quantitative Sensory Testing (QST) derived sensitization measures and activity-related pain predict knee OA pain experiences collected via smartphone ecological momentary assessment (EMA).

**Design:**

Individuals with knee OA were recruited from an urban community in New Zealand. Those eligible to participate underwent baseline QST with clinical measures of activity-related pain also being collected. The knee OA pain experience was collected via smartphone EMA three times daily for two weeks. Mixed effects location scale models were developed using a multilevel modelling approach.

**Results:**

Eighty-six participants with knee OA participated in the study. Mean age was 67.3 years, with most of the participants being female (64%) and New Zealand European (90.6%). Activity-related pain predicted worse and more variable pain intensity, pain interference, and bothersomeness outcomes within and between individuals with knee OA. Widespread cold hyperalgesia and local mechanical hyperalgesia were shown to predict higher within-person variability in pain intensity and pain interference respectively, while mechanical temporal summation predicted less within-person variability in pain intensity and interference.

**Discussion:**

Those demonstrating activity-related pain and sensitization could be at risk of experiencing worse and more variable knee OA pain in the subsequent weeks. Testing for sensitization in clinical practice could therefore identify those at greatest risk of higher and more variable knee OA pain experiences and in greatest need of treatment. Larger validation studies are required, which include individuals with more severe knee OA.

## 1 Introduction

Nervous system sensitization is defined as an ‘increased responsiveness of nociceptive neurons to their normal input, and/or recruitment of a response to normally subthreshold inputs’ [1]. These changes have been demonstrated via Quantitative Sensory Testing (QST) in those with knee OA and include local and widespread pressure hyperalgesia, widespread cold hyperalgesia, heightened temporal summation (TS) and impaired descending modulation [2]. These QST measures have also demonstrated relationships with activity-related pain in those with knee OA [[3], [4], [5]]. Therefore, activity-related pain may also be a measure of nervous system sensitization which demonstrates ecological validity to living and functioning with painful knee OA [[3], [4], [5]]. Nervous system sensitization (hereafter referred to as ‘sensitization’) has significant clinical consequences in individuals with knee OA. Cross-sectionally, this includes greater pain intensity, disability and poorer quality of life on recall-based questionnaires [6,7]. Prospective studies have also been completed, demonstrating the predictive role of sensitization on worse knee OA outcomes as well as treatment responsiveness [5,8,9].

Knee OA pain outcomes are traditionally collected by asking participants to recall their average pain intensity. However, solely considering recalled averages has the potential to overlook the dynamic, fluctuating nature of symptoms in individuals with painful knee OA [10,11]. An assessment method that may better and more accurately capture the knee OA pain experience is Ecological Momentary Assessment (EMA) [12,13]. A defining characteristic of this research method is that measures are repeated multiple times daily to reveal patterns of transient, fluctuating symptoms such as pain, fatigue and mood in real-time, real-life contexts [10,12,13]. Because EMA involves repeatedly measuring the dynamic pain experience over time, within-person variability can be explored [11]. This may reflect a different, yet clinically important aspect of the knee OA pain experience with greater variability in pain intensity being linked with disability and poorer quality of life [11,14].

Few studies have used EMA methods to specifically explore knee OA populations. In the studies that have been published, relationships between self-efficacy and physical activity [15], pain and fatigue [16], fatigue and physical activity [17], pain flare-ups and physical activity [18] have been explored. To the best of our knowledge, whether sensitization (including activity-related pain) can predict within- and between-person pain outcomes collected via EMA in those with knee OA are yet to be explored. Developing a better understanding of the relationship between underlying pain mechanisms, and the knee OA pain experience could improve our understanding and guide treatments to improve outcomes.

Therefore, the aim of this study is to determine whether sensitization, including activity-related pain, predicts prospective pain experiences measured via smartphone EMA. It is anticipated that those demonstrating greater sensitization will demonstrate a worse, more variable pain experience.

## 2 Methods

### 2.1 Study design

**Fig. 1:**
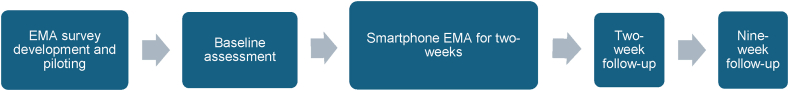
Overview of stages in the U-KOPE study.

The U-KOPE study is a prospective cohort study including smartphone EMA which was completed in Dunedin, New Zealand. A summary of the U-KOPE study is presented in Fig. 1. This study was developed in consultation with the CHecklist for critical Appraisal and data extraction for systematic Reviews of prediction Modelling Studies (CHARMS), the Checklist for Reporting Ecological Momentary Assessment Studies (CREMAS) and EMA literature [19,20]. Ethical approval was obtained through the New Zealand Central Health and Disability Ethics Committee (21/CEN/89). Prior to commencing, the study was also registered in the Australian New Zealand Clinical Trials Registry (Trial ID: ACTRN12621000930886).

### 2.2 Participants

Participants were eligible for inclusion if aged 45–85 years with a diagnosis of knee OA and have experienced daily knee pain for at least three months. Participants fulfilling NICE guidelines for a clinical diagnosis of knee OA were also included (>45 years of age, pain during functioning and morning stiffness lasting no longer than 30 ​min) [21].

Participants were excluded if they were non-English speaking, unable to use a smartphone, had an autoimmune condition or other forms of inflammatory arthritis, had uncontrolled hypertension, skin conditions, lower limb sensory loss, pregnant or within six months postpartum, had undergone or were scheduled for total knee arthroplasty, were recovering from a separate lower limb injury, had a neurological condition, impaired cognition or psychiatric illness (excluding stress and mild to moderate anxiety or depression).

Participants were recruited between May 2021 and January 2022 in Dunedin, New Zealand within hospital outpatient settings and the community through advertisements in local newspapers, health practices and online. At the completion of the study, participants were provided with a $100 supermarket voucher to recognise any costs involved with participating.

### 2.3 Baseline assessment

Eligible participants attended a 90-min baseline assessment at the University of Otago, Dunedin. The following measures were collected at the baseline assessment.

#### 2.3.1 Demographic measures

Participant characteristics, including demographic information (age, sex, ethnicity, knee OA duration, educational level, residential address, and work status) were collected, and anthropometrics (height and weight) were recorded to calculate BMI (kg/m^2^). The Montreal Cognitive Assessment (MoCA), a reliable and valid tool for detecting mild cognitive impairment, was administered to participants at the beginning of the assessment [22]. In participants scoring less than 16, testing was discontinued and these participants were excluded from the study.

#### 2.3.2 Measures of sensitization

Established and standardised QST procedures were completed to quantify aspects of sensory processing and sensitization. Selected QST procedures demonstrate acceptable psychometric properties for assessing abnormal somatosensory processing in those with knee pain [23]. ‘Bedside’ QST procedures were also completed, with these measures being correlated with laboratory-based QST [24]. The following QST measures were performed.

• **Cold pain intensity (CPI)** was assessed using an ice cube which was placed on the participant's non-dominant wrist followed by the affected knee for 10 ​s each. Immediately following each trial, participants reported their greatest pain intensity on a 101-point NPRS [25]. Two trials at each site were performed with the average being calculated. This bedside QST procedure is valid and reliable, significantly correlating with laboratory-based testing thus inferring a bedside measure of cold hyperalgesia [25].
• **Pressure Pain Threshold (PPT)** was measured at the affected medial knee, tibialis anterior (TA)(5 ​cm below the tibial tuberosity) and at the non-dominant wrist [2,6]. A handheld pressure algometer (Wagner Instruments, Greenwich, Connecticut) with a probe area of 1 ​cm^2^ was used at a ramp of 50 ​kPa (kPa) per second [26]. Participants were asked to indicate the moment that pressure sensation became painful. The average across three trials was calculated [26]. Up to five trials were completed at each site if outlier (>50 ​kPa) readings were collected.
• **Punctate Pain Intensity (PPI):** PPI was assessed using a 300-g nylon monofilament (Baseline Evaluation Instruments, NY, USA) over the patella of the affected knee and at the non-dominant wrist [25]. The nylon monofilament was applied perpendicular to the skin at each testing site with enough force to bend the filament. Immediately following each trial, participants reported their pain intensity on a 101-point NRPS. The average across three separate trials was calculated.
• **Mechanical Temporal Summation (MTS)** was assessed over the patella of the affected knee and at the non-dominant wrist [27]. Pain ratings using a 101-point NPRS were recorded following a single 300-g nylon monofilament (Baseline Evaluation Instruments, NY, USA) stimulus [28]. Subsequently, 10 consecutive stimuli were applied at a rate of one stimulus per second within a 1 ​cm^2^ area of skin. After the final stimuli, participants reported their peak pain intensity. The difference between the first pain rating and the peak pain rating was used to calculate MTS [28]. Three separate trials were performed at each site. Continuous MTS scores were used in statistical models.
• **Conditioned Pain Modulation (CPM)**, a measure of descending pain modulation, was examined as per a previous study [29]:
  ◦ Conditioning stimulus: Participants were asked to submerge their dominant hand in a manually circulated 10-degree Celsius ice bath for 2 ​min or until intolerance [30].
  ◦ Test stimulus: PPT40 (PPT with participants indicating when their pain reaches an intensity of 40/100 on the NPRS) was assessed at the non-dominant forearm before and at 30, 60 and 90 ​s following the conditioning stimulus.

Percentage change scores from baseline were used in statistical models with positive change indicating less efficient CPM [29].

• **Movement-Evoked Pain and Sensitivity to Physical Activity:** clinical measures of activity-related pain were also collected. Performance-based tests included a 6MWT and a 30sCST which have been used in previous studies exploring activity-related pain [3,31].
  • 6MWT procedure [31]: A 10-m stretch of hallway was marked with cones placed at each end. Participants were asked to walk around the cones as quickly as possible for 6 ​min to cover as much ground as possible while maintaining safety. Encouragement was provided, with symptom ratings collected at each minute. Rest periods which could include the use of a chair were allowed, although, these were included in the time. The researcher monitored time while counting the total distance walked during the test [31].
  • 30sCST procedure [31]: Participants were seated in a chair (seat height: 42 ​cm) with their feet flat on the floor at shoulder width apart. They also had their arms crossed over their chest. Participants were asked to stand completely before sitting completely. This was repeated as quickly and as safely as possible, for 30 ​s with the researcher counting the total number of full chair stands [31].

Knee discomfort ratings were collected on a 101-point discomfort rating scale (0 ​= ​no discomfort, 100 ​= ​extreme discomfort) before, during (6MWT only) and after each test [3]. MEP representing the average level of pain experienced while undergoing performance-based testing was calculated by taking the average of the discomfort ratings across the 6MWT [32]. SPA was calculated as the difference between prior and peak discomfort ratings [3]. Activity-related pain measures potentially provide a more ecologically valid measure of TS, with links to central sensitization, greater pain intensity and reduced functioning [3,5].

### 2.4 Smartphone ecological momentary assessment

#### 2.4.1 Development and piloting

A smartphone EMA survey was developed following a review of the literature before being piloted on five volunteers with knee OA (Table 1) [[33], [34], [35]]. Volunteers provided their overall feedback, as well as feedback on survey usability, question clarity and potential burden. Feedback collected during smartphone EMA piloting was incorporated into the final EMA survey design.

**Table 1:** U-KOPE EMA survey items and schedule.

EMA Question	Morning	Day	Evening	Response
What is your level of pain right now?	✓	✓	✓	11-point NRS (0 ​= ​No pain, 10 ​= ​Worst pain imaginable)
How much is your pain interfering with what you are doing right now?	✓	✓	✓	11-point NRS (0 ​= ​No interference, 10 ​= ​Totally interfering)
How bothersome is your knee pain currently?	✓	✓	✓	5-item ordinal scale (Not at all, Slightly, Moderately, Very Much, Extremely)
Have you experienced an osteoarthritis flare-up today (‘ … different from usual state … worsening of pain, swelling, stiffness which impacts on sleep, activity, functioning and psychological aspects … ’)?			✓	Yes/No

*Note.* NRS, Numeric Rating Scale.

#### 2.4.2 Smartphone EMA methods

**Fig. 2:**
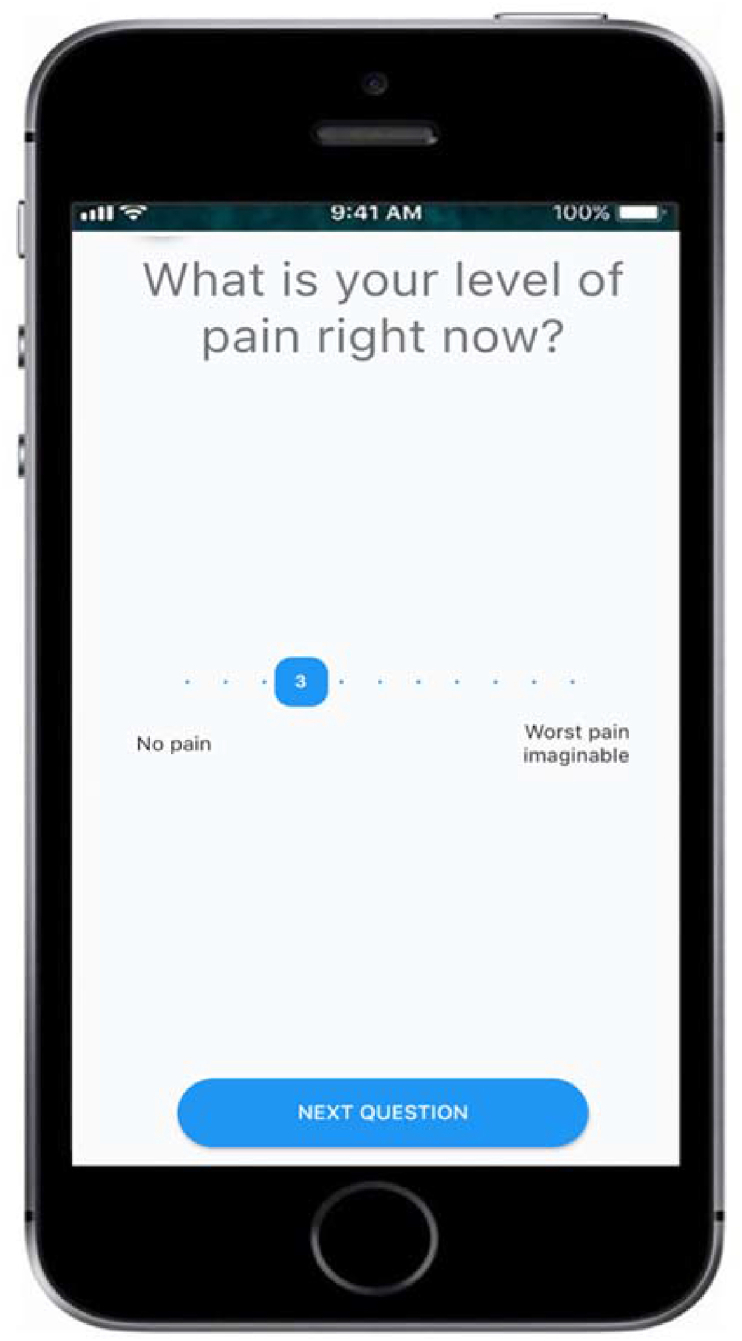
Screenshot of the smartphone EMA survey using the m-Path application.

Following the baseline assessment, eligible volunteers participated in 14 consecutive days of smartphone EMA monitoring (one wave; 10 weekdays and four weekend days). This was reported as the average EMA duration in a recent systematic review exploring the use of EMA in chronic pain research [10]. Participants were required to complete a brief smartphone survey assessing aspects of the knee OA pain experience three times daily using the freely available m-Path application (Table 1) [36]. Participants were either provided with a smartphone or could choose to use their own device and download the freely available m-Path application (see Fig. 2). The researcher-provided smartphone was a Nokia 2.3, Nokia Corporation which used an Android 12 operating system (Snow Cone). In participants choosing to use their own smartphone, there were a range of devices and operating systems used.

At the end of the baseline assessment, participants underwent 10–15 ​min of EMA training with the primary researcher to aid in familiarising themselves with the smartphone, the m-Path application, and survey questions. Participants were also provided with an instructional handout and then signed a statement of commitment.

EMA prompting occurred in a random-stratified manner, with participants receiving an in-app alert randomly within three pre-specified time blocks throughout their day. These time blocks ensured that symptoms after waking in the morning, during the day and before going to bed in the evening were collected to get a representative dataset. To account for variations in daily routines, scheduling considered usual wake and bedtimes reported on the Pittsburgh Sleep Quality Index (PSQI) [37]. The random-stratified blocks were scheduled as follows.

• Morning: A 2-h block was placed immediately after usual wake time.
• Day: A 5-h block was placed from 11 a.m. (or 2 ​h following usual wake time).
• Evening: A 2-h block was placed immediately before usual bedtime.

Therefore, each participant was sent 42 surveys for completion over the duration of the EMA monitoring period. To improve compliance and reduce burden, in-app reminder prompting after 30 ​min as well as ‘snooze’ features for up to 60 ​min were incorporated [19]. In addition, questions regarding symptoms were momentary to reduce recall and improve data quality. Survey question ordering differed between morning, afternoon and evening surveys with prior responses not being viewable.

### 2.5 Data collection and analysis

#### 2.5.1 Data management

The EMA survey administration and data collection were managed by the primary researcher using m-Path [36]. Incoming EMA data was monitored, and participants were contacted if two consecutive assessments were missed. Raw data was exported to Microsoft Excel for cleaning and reformatting. Data cleaning and analysis were not performed until all participants had completed the study.

##### 2.5.1.1 Data processing and reduction (if any)

Reasons for loss to follow-up were recorded with these participants being compared with those completing the study to highlight any potential differences. Latency was less than 60 ​min with responses beyond an initial 60-min window being considered missing. Missing data trends for EMA data were analysed to determine patterns of missingness. Due to the primary method of EMA data analysis being robust to data missing at random (MAR), imputation was not completed. EMA attrition and completion rates (average, SD) as well as systemic differences in completion rates (i.e., demographic variables) were calculated to explore the likelihood of data being MAR. A lack of relationship demonstrated between data missingness and demographic and EMA variables, would support data being MAR.

#### 2.5.2 Statistical analysis plan

A series of multilevel mixed-effects location scale models (MELS) were performed using Mixed models With Intensive Longitudinal Data (MixWILD), Version 1 [38]. The hierarchical nature of the data (multiple observations within multiple participants), allowed for the assessment of within-person location (mean) and scale (variability) effects. The between-person estimate represents a participant's average level relative to the group mean for that variable over a two-week period. The within-person estimate represents participants' momentary ratings relative to their own individual mean over a two-week period [39].

MELS models were developed using MixWILD to explore the effect of sensitization measures on within-person and between-person variability in pain intensity, interference and bothersomeness outcomes. Baseline predictors include QST and activity-related pain measures. The primary time-variant outcomes include pain intensity, interference and bothersomeness scores and the secondary outcomes include the time-invariant flare-up frequency.

Models with random scale were interpreted to account for heterogeneity in location and scale across the knee OA population and improve model convergence. With this being an exploratory analysis, a sample size calculation was not performed. Standardisation of covariates via z-scoring and then the interpretation of models with scale parameters was completed if the models were unable to converge. MELS models were adjusted for three covariates (age, sex and BMI) based on an *a priori* review of the literature and for model simplicity. The level of error considered acceptable for statistical significance was set at p ​≤ ​0.05.

To analyse time-invariant outcomes collected via EMA (OA flare-up frequency) multiple adjusted linear regressions each including one sensitization predictor were completed using SPSS (Version 28.0.1.0). Models were adjusted for covariates including age, sex, BMI, OA symptom duration, baseline pain intensity and socioeconomic status [5]. Multicollinearity and heteroskedasticity were assessed for each analysis by examining multicollinearity statistics as well as scatterplots of residuals. The Durbin-Watson test was used to assess autocorrelation in the residuals with 1.5–2.5 being deemed acceptable. The level of error considered acceptable for statistical significance was set at p ​≤ ​0.05.

## 3 Results

### 3.1 Participant characteristics

**Fig. 3:**
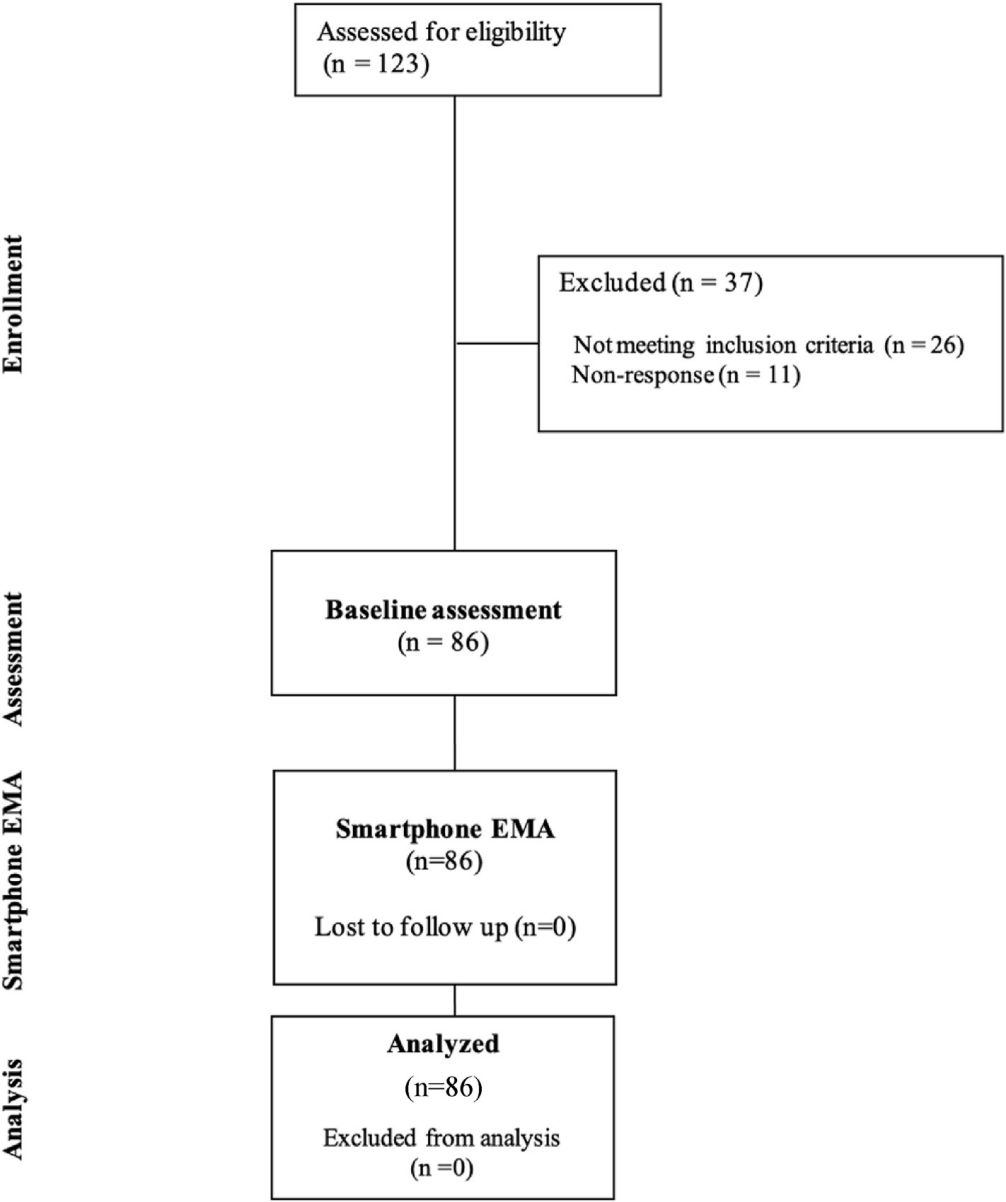
Participant flow diagram.

One hundred and twenty-three individuals registered to participate in the study. Twenty-six (21.1%) did not meet the stated inclusion criteria and 11 participants (8.9%) did not respond to contact attempts from the research team. A final sample of 86 participants were included in the current study, with no loss to follow up. The participant flow diagram is presented in Fig. 3.

Characteristics of study participants are presented in Table 2.

**Table 2:** Characteristics of included participants.

Characteristic	Value^a^
Age (years)	67.3 ​± ​9.1
Sex	Female: 55 [64]
	Male: 31 [36]
Ethnicity	NZ European: 78 [90.6]
	New Zealand Māori: 4 [4.7]
	Indian: 2 [2.3]
	English European: 1 [1.2]
	Egyptian: 1 [1.2]
BMI (kg/m^2^)	32 ​± ​6.8
Waist Hip Ratio	Normal: 1 [1.2]
	Overweight: 19 [22.1]
	Obese: 66 [76.7]
Handedness	Right: 80 [93]
	Left: 6 [7]
Knee OA Duration (years)	9.2 ​± ​9.1
Bilateral OA	Yes: 48 [55.8]
	No: 38 [44.2]
Worst knee	Right: 46 [53.5]
	Left: 40 [46.5]
Highest level of education	No formal qualification: 12 [14]
	Year 10: 1 [1.2]
	Year 13: 12 [14]
	Trade/apprenticeship: 7 [8.1]
	Certificate/diploma: 19 [22.1]
	University degree: 19 [22.1]
	Postgraduate degree: 16 [18.6]
Work status	Full-time employed: 21 [24.4]
	Part-time employed: 9 [10.5]
	Self-employed: 7 [8.1]
	Homemaker: 1 [1.2]
	Retired: 47 [54.7]
	Unable to work: 1 [1.2]

*Note.* BMI, Body Mass Index; kg, kilograms; m, metres; OA osteoarthritis.

a Data are presented as mean ​± ​standard deviation or number [%].

### 3.2 EMA outcomes

Average compliance across the two-week monitoring period was 90.7% ​± ​8.8, meaning participants completed an average of 38.1 out of a possible 42 momentary reports over the EMA period. Eighty participants (93%) used their own phones, while the remaining were loaned and trained with a smartphone provided by the research team. All participants completed the study; therefore, comparisons between participants who completed the study versus those who withdrew were not completed.

Non-parametric correlations (Kendall's Tau and Spearman's) were completed to explore whether compliance was related to demographic variables. Non-response was deemed to be MAR with no evidence of relationships between data missingness and variables including pain intensity (p ​= ​0.43), pain interference (p ​= ​0.97), fatigue (p ​= ​0.42), negative affect (p ​= ​0.51) and the number of flare-up days (p ​= ​0.88).

### 3.3 Sensitization measures

A summary of sensitization measures collected at baseline are presented in Table 3.

**Table 3:** Summary of sensitization measures.

Sensitization measures	Value^a^
PPI (NPRS 0–100)	Knee: 16.2 ​± ​19.1
	Wrist: 10.1 ​± ​12.2
MTS (NPRS 0–100)^b^	Knee: 18.4 ​± ​16.7
	Wrist: 15.6 ​± ​16.8
PPT (kPa)	Knee: 424.8 ​± ​258.4
	Shin: 517.3 ​± ​244.4
	Wrist: 447.4 ​± ​219
CPI (NPRS 0–100)	Knee: 25.6 ​± ​27.8
	Wrist: 21.7 ​± ​27.7
CPM (% change)^‡^	1.6 ​± ​16.2
MEP (NPRS 0–100)	6MWT: 24.4 ​± ​18.8
SPA (NPRS 0–100)	6MWT: 28.2 ​± ​24.2 • SPA: 49 [57] • No SPA: 37 [43]
	30sCST: 22.6 ​± ​25.6

*Note.* MDT, Mechanical Detection Threshold; mN, millinewton; VDT, Vibration Detection Threshold, Hz, Hertz, MFC, medical femoral condyle; PPI, Punctate Pain Intensity; NPRS, Numeric Pain Rating Scale; MTS, Mechanical Temporal Summation; PPT, Pressure Pain Threshold; kPa, kilopascal; CPI, Cold Pain Intensity; CPM, Conditioned Pain Modulation; MEP, Movement-evoked pain; 6MWT, Six Minute Walk Test; SPA, Sensitivity to Physical Activity; 30sCST, 30-s Chair Stand Test.

a Data are presented as mean ​± ​standard deviation or number [%].

b Those reporting a ≥20/100 NPRS change, representing the minimal clinically important difference (MCID) for pain intensity in those with knee OA were classified as demonstrating MTS.

### 3.4 Effects of sensitization on momentary pain outcomes

Effects of sensitization on momentary pain intensity, pain interference and pain bothersomeness outcomes collected via smartphone EMA are presented in Table 4, Table 5, Table 6 respectably below.

**Table 4:** Adjusted mixed models exploring predictors of momentary pain intensity measured via EMA.

Predictor variable	ß	p-value	BS variance (alpha-log)	BS VR	p-value	WS variance (tau-log)	WS VR	p-value	AIC
MEP (6MWT)	**0.05**	**<0.001**	**0.02**	**1.02**	**0.004**	**0.01**	**1.01**	**0.02**	−5358.19
SPA (6MWT)^b^	**0.03**	**<0.001**	0.01	1.01	0.11	**0.01**	**1.01**	**<0.001**	−5785.36
SPA (30sCST)	**0.03**	**<0.001**	−0.01	0.99	0.17	0.01	1.01	0.10	−9307.15
Pain sites	0.08	0.07	−0.06	0.94	0.13	0.05	1.05	0.06	−5372.73
PPT (knee)^a^	−0.27	0.21	−0.16	0.85	0.22	−0.11	0.90	0.29	−5373.94
PPT (wrist)^a^	1.27	0.17	−0.00	1.00	0.19	0.35	1.42	0.36	−4822.15
MTS (knee)	−0.01	0.31	−0.01	0.99	0.24	−0.01	0.99	0.06	−5372.98
MTS (wrist)^b^	−0.01	0.33	0.01	1.01	0.58	**−0.01**	**0.99**	**<0.001**	−5805.34
PPI (knee)^a^	0.01	0.43	0.10	1.11	0.62	0.04	1.04	0.09	−5809.58
PPI (wrist)^b^	0.01	0.54	0.00	1.00	0.92	0.00	1.00	0.29	−5812.76
CPM^a^	0.08	0.69	0.07	1.07	0.70	−0.05	0.95	0.66	−5286.45
CPI (knee)	0.01	0.32	−0.01	1.00	0.35	0.01	1.01	0.11	−5373.81
CPI (wrist)^a^^,^^b^	−0.01	0.96	−0.18	0.84	0.34	**0.07**	**1.07**	**0.01**	−7041.05

*Note.* BS, between-subject; WS, within-subject; VR, variance ratio; AIC, Akaike's information criterion; MEP, movement-evoked pain; 6MWT, 6-min walk test; SPA, sensitivity to physical activity; 30sCST, 30-s chair stand test, PPT, pressure pain threshold; MTS, mechanical temporal summation; PPI, punctate pain intensity; CPM, conditioned pain modulation; CPI, cold pain intensity.

Separate models were completed for each predictor variable adjusted for age, sex and BMI. Findings in bold represent statistical significance (p ​≤ ​0.05).

a Standardised.

b Scale parameters.

**Table 5:** Adjusted mixed models exploring predictors of momentary pain interference measured via EMA.

Predictor variable	ß	p-value	BS variance (alpha-log)	BS VR	p-value	WS variance (tau-log)	WS VR	p-value	AIC
MEP (6MWT)^b^	**0.04**	**<0.001**	**0.02**	**1.02**	**0.01**	**0.02**	**1.02**	**<0.001**	−7240.89
SPA (6MWT)	**0.03**	**<0.001**	**0.02**	**1.02**	**0.00**	**0.02**	**1.02**	**<0.001**	−7467.69
SPA (30sCST)^b^	**0.00**	**0.02**	0.75	2.12	1	**−0.00**	**1.00**	**0.01**	−7200.70
Pain sites^a^	−0.19	0.17	−0.51	0.60	0.30	0.20	1.22	0.30	−5372.73
PPT (knee)^b^	−0.00	0.55	−0.00	1.00	0.86	**−0.00**	**1.00**	**<0.001**	−5373.94
PPT (wrist)^a^^,^^b^	–	–	–	–	–	–	–	–	–
MTS (knee)^a^^,^^b^	−0.02	0.22	−0.05	0.95	0.33	**−0.02**	**0.98**	**<0.001**	−6026.25
MTS (wrist)^a^	−0.03	0.09	−0.06	0.94	0.18	−0.04	0.96	0.06	−6091.14
PPI (knee)^a^	**−0.11**	**0.01**	0.04	1.04	0.91	0.09	1.09	0.62	−6032.09
PPI (wrist)^a^^,^^b^	−0.001	0.97	−0.15	0.86	0.25	−0.03	0.97	0.08	−6036.53
CPM	−0.01	0.37	0.00	1.00	0.88	**−0.00**	**1.00**	**0.03**	−16948.33
CPI (knee)^b^	0.004	0.50	−0.01	0.99	0.20	**0.00**	**1.00**	**0.01**	−6145.72
CPI (wrist)^b^	0.004	0.52	−0.01	0.99	0.39	0.00	1.00	0.09	−6148.52

*Note.* BS, between-subject; WS, within-subject; VR, variance ratio; AIC, Akaike's information criterion; MEP, movement-evoked pain; 6MWT, 6-min walk test; SPA, sensitivity to physical activity; 30sCST, 30-s chair stand test, PPT, pressure pain threshold; MTS, mechanical temporal summation; PPI, punctate pain intensity; CPM, conditioned pain modulation; CPI, cold pain intensity.

Separate models were completed for each predictor variable adjusted for age, sex and BMI. Findings in bold represent statistical significance (p ​≤ ​0.05).

–, unable to converge.

a Standardised.

b Scale parameters.

**Table 6:** Adjusted mixed models predicting momentary pain bothersomeness measured via EMA.

Predictor variable	ß	p-value	BS variance (alpha log)	BS VR	p-value	WS variance (tau log)	WS VR	p-value	AIC
MEP (6MWT)	**0.06**	**<0.001**	−0.01	0.99	0.27	0.00	1.00	0.22	−3031.80
SPA (6MWT)	**0.04**	**<0.001**	−0.00	1.00	0.67	**0.00**	**1.00**	**0.02**	−3035.98
SPA (30sCST)	0.01	0.51	**−0.00**	**1.00**	**0.01**	0.00	1.00	0.57	−3043.01
Pain sites^a^	**0.05**	**<0.001**	–	–	–	–	–	–	−3969.52
PPT (knee)	−0.00	0.46	−0.00	1.00	0.75	−0.00	1.00	0.10	−3043.90
PPT (wrist)	−0.00	0.31	−0.00	1.00	0.77	−0.00	1.00	0.10	−3043.68
MTS (knee)	−0.02	0.21	−0.01	1.00	0.67	−0.00	1.00	0.91	−3044.64
MTS (wrist)	−0.02	0.17	0.01	1.01	0.60	−0.00	1.00	0.39	−3043.81
PPI (knee)	0.01	0.47	−0.01	0.99	0.46	−0.00	1.00	0.92	−3045.13
PPI (wrist)^a^	0.00	0.32	–	–	–	–	–	–	−3983.93
CPM	0.00	0.80	0.01	1.01	0.73	0.00	1.00	0.68	−2996.16
CPI (knee)	0.01	0.37	−0.01	0.99	0.18	−0.00	1.00	0.53	−3044.28
CPI (wrist)	0.01	0.48	−0.01	0.99	0.35	−0.00	1.00	0.68	−3044.97

*Note.* BS, between-subject; WS, within-subject; VR, variance ratio; AIC, Akaike's information criterion; MEP, movement-evoked pain; 6MWT, 6-min walk test; SPA, sensitivity to physical activity; 30sCST, 30-s chair stand test, PPT, pressure pain threshold; MTS, mechanical temporal summation; PPI, punctate pain intensity; CPM, conditioned pain modulation; CPI, cold pain intensity.

Logistic models completed due to ordinal nature of outcome variable. Separate models completed for each predictor variable adjusted for age, sex and BMI.

–, unable to converge.

a Model without random effects.

Greater MEP is associated with greater average pain intensity (β ​= ​0.05, p ​< ​0.001), pain interference (β ​= ​0.04, p ​< ​0.001), pain bothersomeness (β ​= ​0.06, p ​< ​0.001) as well as variability in pain intensity and pain interference between and within subjects. SPA was also related with greater momentary pain intensity (β ​= ​0.03, p ​< ​0.001), pain interference (β ​= ​0.03, p ​< ​0.001), pain bothersomeness (β ​= ​0.04, p ​< ​0.001), between-subject variance in pain interference (alpha (log) ​= ​0.02, VR ​= ​1.02, p ​= ​0.002) as well as within-subject variance in pain intensity and interference. Widespread MTS and CPI also demonstrated significant relationships with within-subject variance in pain intensity (tau (log) ​= ​−0.01, VR ​= ​0.99, p ​< ​0.001 and 0.07, VR ​= ​1.07, p ​< ​0.05 respectively). Local MTS demonstrated an inverse relationship with the within-subject variance of pain interference (tau (log) ​= ​-0.02, VR ​= ​0.98, p ​< ​0.001).

Although models were statistically significant, none of the measures of sensitization met statistical significance for predicting the frequency of knee OA flare-ups (see Table 7).

**Table 7:** Adjusted multivariable models exploring predictors of osteoarthritis flare-up frequency measured via EMA.

Outcome Variable	Predictor Variable^‡^	R^2^	ß	p-value	t	F (p-value – model)
Flare-up frequency	MEP (6MWT)	0.19	−0.15	0.31	−1.02	2.66 (0.02)
	SPA (6MWT)	0.20	−0.12	0.26	−1.13	2.69 (0.02)
	SPA (30sCST)	0.18	−0.03	0.75	−0.32	2.49 (0.02)
	Pain sites	0.18	0.23	0.75	0.32	2.49 (0.02)
	PPT (knee)	0.18	−0.00	0.94	−0.08	2.47 (0.02)
	PPT (shin)	0.18	0.00	0.90	0.13	2.48 (0.02)
	PPT (wrist)	0.20	0.02	0.21	1.26	2.75 (0.01)
	MTS (knee)	0.20	−0.17	0.25	−1.15	2.70 (0.02)
	MTS (wrist)	0.19	−0.12	0.42	−0.81	2.59 (0.02)
	PPI (knee)	0.21	−0.20	0.13	−1.52	2.88 (0.01)
	PPI (wrist)	0.22	−0.37	0.07	−1.85	3.07 (0.01)
	CPM	0.18	−0.06	0.67	−0.43	2.50 (0.02)
	CPI (knee)	0.19	−0.09	0.32	−1.01	2.65 (0.02)
	CPI (wrist)	0.20	−0.13	0.17	−1.38	2.81 (0.01)

*Note.* MEP, movement-evoked pain; 6MWT, 6-min walk test; SPA, sensitivity to physical activity; 30sCST, 30-s chair stand test, PPT, pressure pain threshold; MTS, mechanical temporal summation; PPI, punctate pain intensity; CPM, conditioned pain modulation; CPI, cold pain intensity.

Separate models were completed for each predictor variable adjusted for age, gender, ethnicity, socioeconomic status, baseline pain intensity and BMI.

∗p ​≤ ​0.05; ∗∗p ​≤ ​0.01; ∗∗∗p ​≤ ​0.001.

## 4 Discussion

The key finding of this study is that activity-related pain prospectively predicted worse and more variable pain intensity, pain interference and pain bothersomeness outcomes within and between individuals with knee OA. Widespread cold hyperalgesia and local mechanical hyperalgesia were shown to predict higher intraindividual variability in pain intensity and pain interference respectively. Surprisingly, greater MTS at the knee region predicted less within-person variability in pain intensity and interference.

Pain and reduced functioning are common symptoms reported by those with knee OA which contribute to disability and reduced quality of life [40,41]. In the current study, higher levels of activity-related pain were shown to predict greater pain intensity and interference, including within and between-person variability in these pain outcomes. This means that those with knee OA who experience greater activity-related pain will present with greater overall levels of pain which interfere with daily functioning. Additionally, those with greater pain during activity will present with more fluctuations in their knee OA pain experience. These findings align with previous studies whereby activity-related pain demonstrates cross-sectional and prospective relationships with pain and functional outcomes in those with knee OA [3,5]. Activity-related pain could therefore be a clinically feasible measure of sensitization which doesn't require specialised equipment or training with greater ecological validity to functioning with knee OA. Furthermore, these findings provide a novel contribution to the literature by demonstrating the effects of activity-related pain on variability in knee OA pain experience, which could have important clinical consequences [14].

Activity-related pain also predicted knee OA pain bothersomeness. Pain bothersomeness has been studied in populations experiencing low back pain as well as knee OA to capture the unpleasant, affective dimensions of the pain experience [33]. In those with low back pain, bothersomeness demonstrates criterion validity with pain intensity, disability as well as psychosocial status making it an important measure for the knee OA pain experience [33]. With psychological factors being involved in pain bothersomeness, the moderating role of these psychosocial factors requires consideration.

Some of the QST measures also demonstrated statistically significant relationships with pain intensity and interference. CPI measured at a non-painful site predicted within-person variability in pain intensity whereby a 1-point increase in CPI, predicted 7% greater variability. PPI at the knee was also shown to predict greater average pain interference. These findings align with previous reports confirming those with knee OA demonstrate widespread mechanical and cold hyperalgesia [[42], [43], [44]]. Additionally, those with greater mechanical and cold hyperalgesia have higher levels of pain intensity, worse functioning, poorer quality of life and reduced responsiveness to treatment [[42], [43], [44]]. Therefore, identifying those demonstrating greater sensitivity at a non-painful body part could allow for more targeted, effective treatment to improve knee OA outcomes [43].

In contrast to our hypothesis, MTS was shown to inversely influence the within-subject variability of pain intensity and interference. This may indicate that those with knee OA and greater MTS (a marker of central sensitization), experience a more constant, stable pain presentation with fewer fluctuations. This is supported by studies which have demonstrated that central sensitization predicts the development of greater constant pain over time [45]. However, the average MTS across the sample was low. Additionally, MELS models including MTS with random scale were unable to converge, meaning models with scale parameters were interpreted. This may have resulted in a loss in random scale effects and influenced within-subject findings. Whether this relationship would still be demonstrated if models were adjusted for knee OA duration and psychosocial factors such as pain catastrophizing also warrant consideration.

The Outcome Measures in Rheumatology (OMERACT) group have recently defined an OA flare-up as ‘*a transient state, different from the usual state of the condition, with a duration of a few days, characterized by onset, worsening of pain, swelling, stiffness which impacts on sleep, activity, functioning and psychological aspects that can resolve spontaneously or lead to a need to adjust therapy’* [35]. Contrary to our hypothesis, no statistically significant relationships were demonstrated with the frequency of OA flare-ups. Despite common reports of increased functioning and exercise as antecedents of OA flare-ups [46], studies have also highlighted that OA flare-up triggers are multifactorial with contributions from psychosocial and environmental factors [46]. With models not adjusting for these factors, as well as OA flare-up frequency being significantly higher than in a previous report [47], this may explain the lack of statistically significant findings presented in the current study. Future research should explore multifactorial mechanisms, predictors and consequences of OA flare-ups in those with knee OA in an attempt to better understand and manage these.

Identifying those with knee OA who are at risk of greater pain intensity and variability, as well as pain-related disability could trigger personalized, mechanism-based treatments in an attempt to improve knee OA outcomes [11,48]. Interventions that reduce activity-related pain and sensitization, might also improve the knee OA pain experience [11,48]. With pain variability demonstrating relationships with poorer functioning, sleep quality, work productivity and health-related quality of life; more mild, stable presentations may help those with knee OA develop a greater sense of control, increase their participation in daily activities, and improve their quality of life [11,14]. Treatments may include implementing proactive pain management strategies, tailored physical activity programmes, medication regimes and potentially even psychological coping interventions [14]. Although, further studies exploring whether pain variability can be targeted to improve outcomes are required [48].

This study's findings further support the clinical utility of sensory phenotyping in people with knee OA to improve our understanding of underlying pain sensitization and guide clinical decision-making to improve outcomes [24]. ‘Bedside’ QST could be a more feasible method for assessing sensitization in clinical practice that doesn't require excessive amounts of time or expensive equipment [24]. A recent study confirmed high test-retest reliability of ‘bedside’ QST measures as well as criterion validity when compared with laboratory-based QST [24]. As performed within the current study, assessing pain intensity following the application of an ice cube to a remote, non-painful site or a probe at the knee could identify those with knee OA at greatest risk of a worse, more variable knee OA pain experience [11,48]. However, before ‘bedside’ QST is incorporated into clinical practice, studies are required which explore normative reference values, clinically meaningful cut points and the clinical feasibility of ‘bedside’ QST following the appropriate training of health professionals [24,49].

Strengths of this study included the study design adhering to the CREMAS checklist in an attempt to improve study quality and reduce potential sources of bias [19]. The participants in the current study also had high levels of compliance, improving the accuracy of the data. To the best of our knowledge, this was the first time QST and activity-related pain were explored in relation to EMA outcome data in those with knee OA. A range of measures assessing sensitization were performed including static and dynamic QST, as well as activity-related pain measures which are potentially more feasible in clinical practice. Additionally, relationships with novel pain outcomes collected via EMA such as pain bothersomeness as well as OA flare-ups were explored. A multilevel modelling approach using MELS model was used to analyse the EMA data and allow for the exploration of within-person relationships [38]. As within-person relationships were explored, findings are more applicable and relevant to the individual presenting in clinical practice with pain being a personal, subjective experience [38]. The MELS model also allows for random effects on both the location and scale compared to other multilevel models that solely include random location effects [38].

Limitations of the current study included the sample having a mild knee OA presentation on average who were self-managing in the community. No participants were recruited from the local public hospital orthopaedic outpatient department. The sample demonstrates less sensitization in comparison with knee OA samples reported within the literature [2,3]. This may explain why measures of sensitization didn't significantly predict more knee OA pain experience outcomes. Validation studies with samples including more people with moderate and severe knee OA are warranted. There was also a large degree of variability in knee OA duration, with some participants being newly diagnosed while others had suffered for decades. Scores on psychological measures were also low. Therefore, the sample may not be representative of the wider knee OA population.

Although there were numerous statistically significant findings, the sample and effect sizes were modest meaning these may not being clinically meaningful. MELS models also have a significant computational cost. Therefore, considering technical detail, complexity and practicality in an attempt to improve model convergence was required meaning that only three covariates were included (age, sex, BMI) [38]. Other covariates, such as baseline levels of symptoms, knee OA duration, socioeconomic status and psychological factors may have significantly contributed towards outcomes. Despite simpler models being completed, some failed to converge. This may have been due to the scale in the predictor values, outliers in the data or complexity of the random effects resulting in overfitting. Additionally, the psychometric properties of QST procedures, activity-related pain as well as pain bothersomeness and OA flare-ups measured via smartphone EMA in those with knee OA are yet to be established.

## 5 Conclusion

The current study explored whether sensitization predicted knee OA pain outcomes collected via smartphone EMA. Greater pain experienced during physical activity was shown to predict worse and more variable pain intensity, pain interference and pain bothersomeness within and between individuals with knee OA. Measures of widespread sensitization were also shown to predict higher within-person variability in pain intensity and pain interference respectively. However, MTS was shown to inversely influence the variability of pain intensity and interference indicating that those with greater MTS experience a more constant, stable pain presentation. Testing for sensitization in clinical practice could therefore identify those at greatest risk of worse pain and disability, including more unstable pain presentations. By identifying those at greater risk, treatments can be provided to those in greatest need.

## Author contributions

Mark Overton: Development of study protocol, obtaining ethical approval, recruitment of participants, data collection, analysis, drafting of manuscript.

Nicola Swain: Provided expertise on protocol development, and reviewed drafts of manuscript to improve quality.

Carrie Falling: Provided expertise on protocol development, and reviewed drafts of manuscript to improve quality.

David Gwynne-Jones: Provided expertise on protocol development, and reviewed drafts of manuscript to improve quality.

Roger Fillingim: Provided expertise on protocol development, and reviewed drafts of manuscript to improve quality.

Ramakrishnan Mani: Oversaw protocol development, assisted with data analysis and reviewed drafts of manuscript to improve quality.

## Role of the funding source

Funding for this project was provided by the Otago Medical Research Foundation Jack Thomson grant. The funder had no input into the research design, data analysis, interpretation and overall presentation of findings.

## Declaration of competing interest

All authors declare that they have no conflicts of interest.

## References

[1] International Association for the Study of Pain (2021). Terminology. https://www.iasp-pain.org/resources/terminology/.

[2] Fingleton C. (2015). Pain sensitization in people with knee osteoarthritis: a systematic review and meta-analysis. Osteoarth. Cartil..

[3] Wideman T.H. (2014). Increased sensitivity to physical activity among individuals with knee osteoarthritis: relation to pain outcomes, psychological factors, and responses to quantitative sensory testing. Pain.

[4] Simon C.B. (2021). Static and dynamic pain sensitivity in adults with persistent low back pain: comparison to healthy controls and associations with movement-evoked pain versus traditional clinical pain measures. Clin. J. Pain.

[5] Overton M. (2023). Activity-related pain predicts pain and functional outcomes in people with knee osteoarthritis: a longitudinal study. Front. Pain Res..

[6] Imamura M. (2008). Impact of nervous system hyperalgesia on pain, disability, and quality of life in patients with knee osteoarthritis: a controlled analysis. Arthritis Care Res..

[7] Moore R.L. (2020). The relationship between clinical and quantitative measures of pain sensitization in knee osteoarthritis. Clin. J. Pain.

[8] O'Leary H. (2017). Nervous system sensitization as a predictor of outcome in the treatment of peripheral musculoskeletal conditions: a systematic review. Pain Pract..

[9] Georgopoulos V. (2019). Quantitative sensory testing and predicting outcomes for musculoskeletal pain, disability, and negative affect: a systematic review and meta-analysis. Pain.

[10] May M. (2018). Ecological momentary assessment methodology in chronic pain research: a systematic review. J. Pain.

[11] Schneider S. (2012). Individual differences in the day-to-day variability of pain, fatigue, and well-being in patients with rheumatic disease: associations with psychological variables. Pain.

[12] Shiffman S., Stone A.A., Hufford M.R. (2008). Ecological momentary assessment. Annu. Rev. Clin. Psychol..

[13] Overton M. (2023). Are ecological momentary assessments of pain valid and reliable? A systematic review and meta-analysis. Clin. J. Pain.

[14] Hutchings A. (2007). The Longitudinal Examination of Arthritis Pain (LEAP) study: relationships between weekly fluctuations in patient-rated joint pain and other health outcomes. J. Rheumatol..

[15] Zhaoyang R., Martire L.M., Sliwinski M.J. (2017). Morning self-efficacy predicts physical activity throughout the day in knee osteoarthritis. Health Psychol..

[16] Smith D.M., Parmelee P.A. (2016). Within-day variability of fatigue and pain among African Americans and Non-Hispanic Whites with osteoarthritis of the knee. Arthritis Care Res..

[17] Fawole H.O. (2020). Temporal associations between physical activity, mental activity and fatigue dimensions in knee osteoarthritis: an exploratory intensive longitudinal study. Fatigue Biomed. Health Behavior.

[18] Parry E., Ogollah R., Peat G. (2019). ‘Acute flare-ups’ in patients with, or at high risk of, knee osteoarthritis: a daily diary study with case-crossover analysis. Osteoarth. Cartil..

[19] Liao Y. (2016). A systematic review of methods and procedures used in ecological momentary assessments of diet and physical activity research in youth: an adapted STROBE Checklist for Reporting EMA Studies (CREMAS). J. Med. Internet Res..

[20] Moons K.G. (2014). Critical appraisal and data extraction for systematic reviews of prediction modelling studies: the CHARMS checklist. PLoS Med..

[21] National Clinical Guideline, C. (2014). Osteoarthritis: Care and Management in Adults. 2014, National Institute for Health and Care Excellence (UK).

[22] Nasreddine Z.S. (2005). The Montreal Cognitive Assessment, MoCA: a brief screening tool for mild cognitive impairment. J. Am. Geriatr. Soc..

[23] Alqarni A.M. (2018). Test procedures to assess somatosensory abnormalities in individuals with peripheral joint pain: a systematic review of psychometric properties. Pain Pract..

[24] Koulouris A.E. (2020). Reliability and validity of the Boston bedside quantitative sensory testing battery for neuropathic pain. Pain Med..

[25] Zhu G.C. (2019). Concurrent validity of a low-cost and time-efficient clinical sensory test battery to evaluate somatosensory dysfunction. Eur. J. Pain.

[26] Rolke R. (2006). Quantitative sensory testing: a comprehensive protocol for clinical trials. Eur. J. Pain.

[27] Skou S.T. (2013). Relating clinical measures of pain with experimentally assessed pain mechanisms in patients with knee osteoarthritis. Scand. J. Pain.

[28] Carlesso L.C. (2022). Use of IMMPACT recommendations to explore pain phenotypes in people with knee osteoarthritis. Pain Med..

[29] Mani R. (2019). Sedentary behaviour facilitates conditioned pain modulation in middle-aged and older adults with persistent musculoskeletal pain: a cross-sectional investigation. Pain Rep..

[30] Yarnitsky D. (2015). Recommendations on practice of conditioned pain modulation (CPM) testing. Eur. J. Pain.

[31] Dobson F. (2013). OARSI recommended performance-based tests to assess physical function in people diagnosed with hip or knee osteoarthritis. Osteoarth. Cartilage.

[32] Fullwood D. (2021). Toward understanding movement-evoked pain (MEP) and its measurement: a scoping review. Clin. J. Pain.

[33] Dunn K.M., Croft P.R. (2005). Classification of low back pain in primary care: using “bothersomeness” to identify the most severe cases. Spine.

[34] Carlozzi N.E. (2018). The reliability of end of day and ecological momentary assessments of pain and pain interference in individuals with spinal cord injury. Qual. Life Res..

[35] Guillemin F. (2019). Developing a preliminary definition and domains of flare in knee and hip osteoarthritis (OA): consensus building of the flare-in-OA OMERACT Group. J. Rheumatol..

[36] Mestdagh M. (2022).

[37] Buysse D.J. (1989). The Pittsburgh Sleep Quality Index: a new instrument for psychiatric practice and research. Psychiatr. Res..

[38] Dzubur E. (2020). MixWILD: a program for examining the effects of variance and slope of time-varying variables in intensive longitudinal data. Behav. Res. Methods.

[39] Hedeker D., Dunton G. (2018).

[40] Cui A. (2020). Global, regional prevalence, incidence and risk factors of knee osteoarthritis in population-based studies. EClinicalMedicine.

[41] Leifer V., Katz J., Losina E. (2022). The burden of OA-health services and economics. Osteoarth. Cartilage.

[42] Bevilaqua-Grossi D. (2019). Thermal and mechanical pain sensitization in patients with osteoarthritis of the knee. Physiother. Theory Pract..

[43] Wright A. (2017). Cold pain threshold identifies a subgroup of individuals with knee osteoarthritis that present with multimodality hyperalgesia and elevated pain levels. Clin. J. Pain.

[44] O'Leary H. (2018). Pain sensitization associated with nonresponse after physiotherapy in people with knee osteoarthritis. Pain.

[45] Aoyagi K. (2021). Development of a pain sensitivity index to examine the transition from intermittent to constant pain in knee osteoarthritis: the multicenter osteoarthritis study. Osteoarth. Cartilage.

[46] Thomas M.J. (2021). Triggers for acute flare in adults with, or at risk of, knee osteoarthritis: a web-based case-crossover study in community-dwelling adults. Osteoarth. Cartilage.

[47] Bowden J.L. (2021). Best-practice clinical management of flares in people with osteoarthritis: a scoping review of behavioral, lifestyle and adjunctive treatments. Semin. Arthritis Rheum..

[48] Madden V.J. (2021). Variability in experimental pain studies: nuisance or opportunity?. Br. J. Anaesth..

[49] Reimer M. (2020). Sensory bedside testing: a simple stratification approach for sensory phenotyping. Pain Rep..

